# Our April Number

**Published:** 1896-04

**Authors:** 


### EDITORIAL.

### OUR APRIL NUMBER.

In sending forth this issue of the Journal among the entire veterinary profession of North America, we present to its readers the most complete publication of its kind that has ever been issued. With one hundred and more compactly printed pages of reading matter of varied interest to each member of our calling, we hope and trust that this missionary effort will sow good seed that shall ripen in the interests of stronger, better, and higher journalism for our profession than has yet been offered to so large a class of readers.

We present in conjunction with this issue a larger number of *bona fide* pages of advertisements than has ever before been published in a single number, not one of which but what our readers will find of the highest integrity and character in business circles. Their faith in the future of the veterinary profession is best attested by their generous use of our pages in this number, and they thus contribute to veterinary journalism in seeking for their products a wider field, which we are sure will be mutually beneficial. We are grateful to our advertisers for their assistance.

To the profession that has so generously come to the support of the Journal with the new year, we most heartily tender our sincere thanks and appreciation. We believe the new era of journalism in our profession is at hand, and that the increased attractions of our contemporaries, the *Review* and *Magazine*, will bring to them a more generous recognition and support, which they merit and should command. The Journal has only one aim to serve, to LEAD VETERINARY JOURNALISM IN AMERICA In the future, as in the past, no time or money will be spared to attain this end, that our profession may be made broader, better, and stronger, and fill a more exalted place in the needs of our people; where they may create for man the greatest safeguards in the pathway of our food-supply and contribute to the animal creation the tenderest care, the greatest relief from suffering, and the protection from dangers of fashion and service that an all-wise Providence has designed through the veterinary profession for their protection.

